# American Academy of Dental Science

**Published:** 1898-01

**Authors:** 

**Affiliations:** American Academy of Dental Science


### AMERICAN ACADEMY OF DENTAL SCIENCE.

The regular monthly meeting of the American Academy of Dental Science was held at Young's Hotel, Wednesday evening, May 5, 1897, at six o'clock. President Andrews in the chair.

Subject for discussion was "Replantation of Teeth."

*Dr. Perrin.*—The operation of replantation, though rarely required, is one that should be familiar to every dentist, for occasionally a case presents itself in which no other treatment will effect so rapid a cure.

I have had several cases where replantation was resorted to, with such happy results that I feel prompted to state them at this time. In each of these pericementitis was present to such a degree as to cause unbearable pain; removal of the filling or perforation of the tooth was impossible. My method has been, after extracting the tooth, to remove the filling, cleanse and disinfect the pulp-cavity and root-canals, and fill the same with the usual materials. I also remove the inflamed portion of the pericementum and grind away about one-sixteenth of the apex of the root. The socket is then washed out with warm water, being careful to remove all clotted blood. The tooth is then pressed firmly into place, and perfect adaptation results in about a week.

My first case was in May, 1884. A man about thirty-five years of age, with a left superior bicuspid affected with periodontitis. The tooth was extracted and replaced, and has served well for over ten years. My second case was in November, 1886, also a left superior bicuspid, very sore to the touch, and containing a large amalgam filling, the removal of which was impossible. The tooth was extracted and replaced, and in less than a week was as useful as ever. It is still perfectly firm and useful.

Case III.—December, 1890. A young man twenty-two years old. First right inferior molar in same condition as the preceding cases. This was extracted and replaced, and, although it was necessary to grind the roots quite extensively to make them parallel, the tooth soon became firm and healthy, and is now in good condition.

Case IV.—November, 1892. A lady brought in her hand her second left superior molar carefully tied up in paper, it having been extracted by mistake while removing the third molar. This was replaced, and is to-day a useful tooth.

*President Andrews.*—How many cases have you had in all?

*Dr. Perrin.*—Only these four that I have record of. I have had several others the results of which are unknown.

*Dr. Cutter.*—Did you say you simply washed out the socket with warm water?

*Dr. Perrin.*—Yes; but I am sure to get out all particles of loose tissue and scrape off the reddened pericementum from the root.

*Dr. Bradley.*—May I ask Dr. Perrin if in any of these cases there was an established fistula?

*Dr. Perrin.*—In none of these cases had there been an abscess formed; there was simply a condition of pericementitis.

*Dr. Stevens.*—I had a case where I removed a right central incisor to relieve an alveolar abscess, a blind abscess that was very painful. I removed the tooth, cleansed the socket of the abscess, thoroughly sterilized the socket and the tooth, filled the canal, and returned the tooth to its place. The patient in two days was entirely over the effects of the operation, and the tooth has never given further trouble.

*Dr. Bradley.*—Did you wire the tooth in place?

*Dr. Stevens.*—I did, but removed the ligature after two days.

*Dr. Allen.*—I have had three of these cases altogether. The first was that of a young woman who came to me suffering from a severe chronic alveolar abscess with a fistula over the left superior cuspid. Another dentist had previously treated the tooth, but to be able to work intelligently I removed the filling, and found the canal (which had been filled with gutta-percha) free from odor and perfectly sealed to the apical foramen. After opening the foramen and treating the case several days, I was surprised to find that my patient experienced no relief. I then decided to extract the tooth, to which proposition the patient readily assented. Upon removing the tooth, I discovered the cause of the trouble to be a sharp spur of calculus extending at right angle from the root, at a point fully an eighth of an inch from its apex. I removed the calculus, polished the roughened surface (there being no evidence of necrosis), and returned the tooth to its socket. It was my pleasure to examine the case two weeks ago and to find the tooth apparently as firm as any normal tooth, having stood a three years' test.

Another case was that of a lady sixty years of age. The tooth in this instance was the right inferior sixth-year molar, which I treated in the same manner as described by Dr. Perrin. A year later it was doing well, but I have had no report from the patient of late, though it is likely I should have been informed had any unfavorable symptom developed.

The third case was that of a gentleman for whom I crowned the left superior first bicuspid. In drilling out the canal for the pin of the crown I accidentally encroached upon the bifurcation of the root; this opening was sealed as well as possible with gutta-percha and the crown adjusted. The tooth remained comfortable for six months or so, when it began to show signs of suppuration. Knowing well the cause of the trouble, I extracted the root and the crown together, filled the perforation in the root with gold, and then replaced the root in its socket. The gentleman is now in Europe, and but recently sent me word that the tooth was still doing good service.

*Dr. Adams.*—I have had some experience in this line. One case in particular which I remember was that of a lateral incisor which had an alveolar abscess that had been discharging for three years. I could not cure the case, so I extracted the tooth and cut off the end of the root, which was denuded, and returned the tooth to its socket. It remained in a perfectly healthy condition for years, until the death of the patient. The details of the operation were as follows: The root was filled through a cavity in the palatal surface; this cavity was filled with gold, which was built out beyond the surface, and the ligatures which were to secure the tooth in place were embedded in the filling; this was done to prevent the slipping of the ligature.

Several years after the replanting of the tooth a cavity appeared in the proximal surface, and I wedged the tooth apart and filled it without injury to the tooth.

I find that the length of time during which such teeth will serve is variable. Some of them have, at the end of perhaps seven or eight years, suddenly given out, and I would find that the root was badly absorbed and the tooth had gone completely to pieces. But I think in cases of abscesses which are obstinate it is very good treatment.

*Dr. Eames.*—At a meeting of the Massachusetts Dental Society in 1884 I exhibited a case in which the superior lateral was turned on its axis, so that its mesial surface presented directly forward, and I then explained that I had extracted that lateral, enlarged the socket, and returned the tooth to its proper position in the mouth. I had also another case answering in every particular to the one just described, and treated in the same way. The replanted teeth performed satisfactory service in each case for about ten years, when, as I have been informed, they came out. I thought it rather remarkable that they lasted about the same length of time.

Another case was that of an inferior cuspid, replanted about the year 1877, and which did good service for about fifteen years. Some other cases lasted perhaps not more than six to eight years. Two or three are still giving satisfaction, with a record behind them of eight years. I will simply refer to two cases of recent interest,—one a superior left central which had had a fistula on the labial surface for a number of years. A crown had been put on by a former dentist, which had been recently removed in order to treat the root, and the present dentist had treated it for months without healing the fistula. I saw the case in consultation, and after making a counter-opening and injecting antiseptic fluids freely without good results, I etherized the patient and made a free incision near the apex of the root, removed the tissues there, and with a large new bur amputated the end of the root and burred away the bone in the vicinity. But the fistula still continued to discharge. The last resort was the plugging of this central root (which was a mere shell) with wood and extracting it. It was found that on the palatal surface of the root was an opening from the canal which had been drilled through, and that filling-material had been stuffed through this opening plentifully. It was in such a position that it would have been impossible to get at it by any reasonable surgical interference without extracting the root. After curetting the surface thoroughly and washing the cavity with bichloride of mercury solution, I prepared the root and returned it to its socket. A plate which was being worn kept it in position, overcoming its tendency to come out. This was done about six weeks ago, and the tooth is now doing very well, being fully as firm in its socket as before being treated.

In another recent case there was violent inflammation with swelling of the face and gum, and a fistula from a superior right cuspid. On account of the great pain the tooth was extracted, and a large sac was found at the end of the root. This was removed with a portion of the root and the tooth returned to its socket. This tooth had a strong tendency to come out; holding it in place for a long time with the fingers was not sufficient to keep it there, I therefore ligated a wire to the second bicuspid and passed it over the cuspid and ligated it to the lateral. The implanted tooth was then tied down to the wire, which held it in place. It now appears to be as serviceable as any tooth.

*Dr. Robinson.*—I had a case, I think it was in 1873 or 1874. A gentleman came into my office with a tooth that was aching and wanted it extracted. Examination showed a cavity which extended nearly to the pulp. I wanted him to allow me to destroy the pulp and fill the canal, but he was impatient and would not have it done, so I was obliged to extract the tooth. I told him I thought it was too bad to take out a good tooth like that and break up the arch, and I suggested filling it and replacing it, and this he agreed to. I have seen him within two years, and he said that it was the best tooth in his head. I have had several other cases that turned out very nicely.

*President Andrews.*—Perhaps Dr. Preston will tell us of his experience in this line.

*Dr. Preston.*—I have extracted several teeth and filled them and put them back again, but I cannot now recall one that ever amounted to anything. The roots all became rough and looked worm-eaten. I filled one for a young boy away back in 1839. In those days you could not destroy the pulp as you can now, and it was necessary either to extract the tooth and put it back, or keep it out altogether.

*Dr. Belyea.*—What did you fill the roots with?

*Dr. Preston.*—I did not fill the roots at all, but filled the external cavity with tin-foil. I found one man who seemed to be well pleased with the result of the operation. I asked him about the tooth several years after replacing it, and he said that he had never had any trouble from it, and if any one wanted to know about such an operation to send them to him. The most peculiar case of replantation I ever heard of was that of a lady for whom I extracted a tooth as far back as 1842. She felt badly about having it extracted, and she afterwards told me that when she got home she took an iron spoon and melted some lead, washed the tooth thoroughly, carefully poured the lead into the cavity and put the tooth back in her head. I do not know how long it lasted her.

*Dr. Werner.*—We have only heard of favorable cases of replantation; while I could perhaps report two that were favorable, I have distinct recollections of two unfavorable cases. In twenty years' practice I have had perhaps six or seven cases, two of which could perhaps be considered favorable, but two others were lamentable failures. I do not consider my experience to be entirely successful, and only in extreme cases do I consider the operation justifiable. I should not think of replanting a root in a diseased socket. I had a case recently where I thought of replanting, and would have done so if I had felt fairly certain of the result. In resetting a bridge I was obliged to remove one of the roots, a central, which was perhaps one of the best of the supporting teeth, and I wished that I could have felt justified in replanting that root.

*President Andrews*—I recall two cases of abscessed teeth, where I extracted the teeth, carefully filled the roots, dressed them down, and replaced them in their sockets; I noticed that in a year or two they became solidly fixed to the jaws, calcified so solidly that in tapping them they appeared to be a part of the jaw. It seemed as if the periosteal tissue about the teeth had completely calcified, and I think you will find that in most cases of replanted teeth on tapping them they give the impression that they have become united by calcification to the alveolus. In my cases the teeth began to loosen in a few years, and the roots seemed to be nearly absorbed through the action of the cells which we call the osteoblasts. Both cases had a similar history.

William H. Potter, D.M.D.,
*Editor American Academy of Dental Science.*

